# Wound closure after total knee arthroplasty: Comparison of polypropylene and polyglactin 910 suture-a randomized controlled trial

**DOI:** 10.12669/pjms.42.8.12048

**Published:** 2026-08

**Authors:** Mansoor Ali Khan, Nek Mohammad, Muhammad Amin Chinoy, Muhammad Danish Khan

**Affiliations:** 1 Mansoor Ali Khan, FCPS. Department of Orthopedics, Aga Khan University Hospital, Karachi, Pakistan; 2 Nek Mohammad, FCPS. Department of Orthopedics, Indus Hospital and Health Network, Korangi, Karachi, Pakistan; 3 Muhammad Amin Chinoy, FRCS, Department of Orthopedics, Indus Hospital and Health Network, Korangi, Karachi, Pakistan; 4 Muhammad Danish Khan, FCPS. GDA Pak-China Friendship Hospital (Under the management of Indus Hospital and Health Network), Makran, Balochistan, Pakistan

**Keywords:** Erythema, Osteoarthritis, Polyglactin 910, Polypropylene, Post-operative complications, Surgical site infection, Total knee arthroplasty, Wound dehiscence

## Abstract

**Objective:**

To compare the outcomes of wound closure using polypropylene and subcuticular polyglactin 910 sutures in patients undergoing total knee arthroplasty (TKA).

**Methodology:**

A randomized, assessor-blinded controlled trial was conducted at the Department of Orthopedics, Indus Hospital and Health Network, Karachi, from February 2022 to February 2025, during the hospital’s Arthroplasty Week. All the patients who had undergone bilateral total knee arthroplasty in this timeframe and were eligible were included. Polypropylene sutures were used to close one knee in each patient and polyglactin 910 sutures were used to close the opposing knee. Correlations between independent and dependent variables were evaluated and regression analysis was performed.

**Results:**

A total of 40 patients were enrolled in the study with a female predominance (67.5%). Post-operative erythema was frequent in the polypropylene group in the early post-operative days while complications were more frequently observed in the polyglactin 910 group during the later postoperative period. Female sex, diabetes mellitus, and American Society of Anesthesiologists (ASA) Grade III were significant factors associated with wound dehiscence and surgical site infection.

**Conclusion:**

There was no statistically significant difference in postoperative complications in patients undergoing total knee arthroplasty between polyglactin 910 and polypropylene sutures. Both sutures can be effectively used for wound closure, but in the resource-limited environment, polyglactin 910 could be a better choice as it is an absorbable material, and the sutures can be removed without it.

***Trial Registration:*** NCT06373900.

## INTRODUCTION

Total knee arthroplasty (TKA) is a highly effective procedure performed in patients with advanced osteoarthritis of the knee to reduce pain, regain knee mobility, and improve the quality of the patient’s life.^1^ With the increasing life expectancy of the world population, increasing obesity, and the expanding indications, TKA is one of the most commonly performed orthopedic procedures around the world.^2^,^3^ The application of advanced surgical techniques and improved methods of perioperative care are associated with even better surgical outcomes and higher procedure success rates.^4^,^5^ Multiple factors, including the condition of the knee joint before surgery, surgical techniques, choice of prosthesis, rehabilitation after surgery, and factors related to the patient such as health status and comorbidities, determine the outcome of TKA.^6^-^8^ Among these factors, proper closure of the surgical wound is one of the most important factors in achieving a good outcome and reducing postoperative complications. This closures also has an indirect impact on the satisfaction of the patient and the outcome of the surgery 9,10

The primary goal of wound closure after TKA is the secure and permanent closure of the wound, and the reduction of complications. These complications include pain, fibrosis, wound dehiscence, infection, slow healing and the need for reoperation.^9^,^10^ Proper selection of the sutures is one of the steps in the process. These sutures can have an impact on the physiological and biological factors that can affect the healing process of the wound.

Polypropylene sutures are non-absorbable synthetic sutures with a high tensile strength, low tissue reactivity and provide long-term support to wounds.^11^ Polyglactin 910 on the other hand is a absorbable synthetic suture made of the polymers of glycolic acid and lactic acid that provide short-term support to wounds and is degradable within the tissue.^12^ It can be fully absorbed, reducing the need for suture removal, and decreasing chronic foreign body response. Despite their common usage, the choice of polypropylene vs. Polyglactin 910 sutures has been a subject of debate among orthopedic surgeons. There is a limited body of literature that support the view that non-absorbable sutures provide better long-term stability of wounds, however there is literature to support that absorbable sutures are associated with a reduced incidence of wound complications and provide better comfort to the patient.^13^ At present, there are a limited number of randomized controlled trials that directly compare the two suture materials for the closure of the wound in TKA. Therefore, this study aimed to analyze the outcomes of the use of the two suture materials in patients who have undergone Total Knee Arthroplasty. The findings of this study would promote orthopedic surgeons to make an evidence based choice of sutures that could lead to improved outcomes for patients in terms of reduce the burden of ongoing post-operative care and improving patients’ quality of life.^14^,^15^

## METHODOLOGY

The present parallel randomized controlled trial was conducted from February 2022 to February 2025 at the Department of Orthopedics, Indus Hospital and Health Network, Karachi, to compare the outcomes.

### Ethical approval:

Approval for the Indus Hospital and Health Network institutional review board was obtained beforehand. (IHHN_IRB_2021_10_004; dated February 4, 2022).

### Inclusion & Exclusion Criteria:

All patients aged 50–80 years, of either sex, diagnosed with end-stage osteoarthritis or post-traumatic arthritis and scheduled for bilateral primary total knee arthroplasty at the Department of Orthopedics, Indus Hospital and Health Network, Karachi, were enrolled in the study. Exclusion criteria included patients having existing skin, neuromuscular, or connective tissue disorders, rheumatoid arthritis, immunosuppression, and morbid obesity. Only patients planned for simultaneous bilateral total knee arthroplasty were included to enable within-patient comparison.

### Intervention:

Wound closure was done in layers in a standardized procedure in all patients undergoing bilateral primary TKA. Absorbable sutures were used to close deep layers, such as arthrotomy and subcutaneous tissues. In skin closure, one knee was randomly allocated to closure with interrupted non-absorbable polypropylene suture whereas the opposite knee was closed with subcuticular absorbable polyglactin 910 sutures (Vicryl Rapide). The subcuticular closure was not augmented with any additional skin sutures or staples. Subcuticular polyglactin 910 is an established method of skin closure in orthopedic surgery, and has been noted in the past research of cosmetic results and wound complications after arthroplasty. A standardized medial parapatellar approach was used to carry out all the procedures by the same surgical team to achieve uniformity.

### Outcomes:

Postoperative wound healing was evaluated at days 3, 7, 15 and 30. Standardized definitions were used to assess wound related complications. The definition of surgical site infection (SSI) was by the criteria of the Centers for Disease Control and Prevention (CDC) which include the following: purulent discharge, localized swelling, erythema, warmth and positive culture of the wound. The definition of wound dehiscence included the partial or complete dislocation of wound edges. Failure of the wound to reach satisfactory epithelialization after 30 days postoperative was used as a definition of delayed wound healing. The validated scale of assessment, the Hollander Wound Evaluation Scale, was used to evaluate wound assessment and cosmetic outcomes, considering parameters like step-off borders, irregularities of contour, margin separation, edge inversion, excessive distortion, and overall cosmetic appearance. The parameters were rated as either 0 or 1 and the overall score was between 0 (poor outcome) and 6 (optimal outcome). Systemic symptoms (not part of the Hollander score) were recorded separately and defined as the presence of fever, malaise, or other systemic signs suggestive of infection during the postoperative period. The simple subjective scale (satisfied vs not satisfied) was used to measure patient satisfaction based on the perception of wound appearance when the patient was at the final follow-up visit.

### Randomization:

A within-patient randomization (paired design) was employed. In patients undergoing bilateral TKA, the allocation of suture type (polypropylene or polyglactin 910) to the right or left knee was determined using computer-generated random numbers. This design allowed each patient to serve as their own control, thereby minimizing inter-patient variability.

### Blinding (Masking):

Outcome assessment was performed in a blinded manner. Patients were not informed about the type of suture used, and postoperative wound evaluation was conducted by an independent assessor who was unaware of the allocation. However, the operating surgeons could not be blinded due to the nature of the procedure.

### Statistical analysis:

Data analysis was performed using SPSS version 24.0. After assessing the normality of the data, all continuous variables were represented as medians (IQR). Discrete variables are shown as frequencies and percentages. An association was evaluated between the type of sutures and the dependent variables. Regression analysis was performed to evaluate the impact of the independent variables on wound dehiscence, wound healing and surgical site infection. For univariate analysis, variables with a p-value of <0.25 were considered for inclusion in multivariate analysis. Statistical significance in multivariate analysis was set at p-value <0.05.

## RESULTS

A total of forty patients were enrolled in this study. There was a female predominance in the present study, with a M:F ratio of 1:2.07 (Table-I). The median (IQR) age of the study participants was 58 years (50.5 - 69.0 years). Hypertension was the most frequent comorbidity [33(82.5 %)], followed by diabetes mellitus [15 (37.5%)]. No significant difference was observed in outcomes based on the side of surgery (right vs left knee), indicating that side allocation did not influence the study results.

**Table-I:** Patient demographic and procedural characteristics.

***Gender (M/F)***	
n (%)	13 (32.5)/27 (67.5)
***Age, years***	
Median (IQR)	58.0 (50.5 - 69.0)
***Height, cm***	
Median (IQR)	159.0 (150.0 – 165.0)
***Weight, kg***	
Median (IQR)	74.5 (65.0 – 82.0)
***ASA score***	
Mean (SD)	1.9 (0.38)
***Comorbidities (n; %)***	
Hypertension	33 (82.5)
Type 2 Diabetes	15 (37.5)
Current or former smoker	3 (7.5)
***Length of incision, cm***	
***Left knee,***	
Median (IQR)	16.0 (15.0 – 18.0)
***Right knee,***	
Median (IQR)	17.0 (16.0 – 19.0)

At baseline, American Society of Anesthesiologists (ASA) Grade II was the most frequently observed class in the study cohort. (85%). The complications and the Hollander score of the patients as observed in the two suture groups are displayed in Table-II. Presentation of erythema was observed in more patients in the polypropylene group versus polyglactin 910 group for postoperative days 3 and 7 i.e., 21 versus 16 and 14 versus 12 respectively but then became vice versa for days 15 and 30 during which the polyglactin 910 group had more patients i.e., five versus three and two versus zero respectively.

**Table-II:** Hollander score and sub-scores as per suture type.

	Polyglactin 910				Polypropylene			
	D3	D7	D15	D30	D3	D7	D15	D30
A1: Erythema (n, %)	16 (40)	12 (30)	5 (12.5)	2 (5.0)	21 (52.5)	14 (35)	3 (7.5)	0 (0)
A2: Wound dehiscence (n, %)	0 (0)	1 (2.5)	1 (2.5)	1 (2.5)	0 (0)	0 (0)	0 (0)	0 (0)
A3: Discharge from wound (n, %)	12 (30)	8 (20)	2 (5.0)	2 (5.0)	10 (25)	5 (12.5)	1 (2.5)	0 (0)
A4: Blisters (n, %)	2 (5.0)	1 (2.5)	0 (0)	0 (0)	2 (5.0)	0 (0)	0 (0)	0 (0)
A5: Warmth (n, %)	11 (27.5)	5 (12.5)	5 (12.5)	2 (5.0)	12 (30)	10 (25)	2 (5.0)	0 (0)
A6: Tenderness (n, %)	6 (15)	3 (7.5)	1 (2.5)	2 (5.0)	9 (22.5)	4 (10)	0 (0)	0 (0)
B1: Stitch abscess (n, %)	0 (0)	1 (2.5)	1 (2.5)	1 (2.5)	1 (2.5)	0 (0)	0 (0)	0 (0)
B2: Cellulitis>1cm (n, %)	9 (22.5)	4 (10)	1 (2.5)	2 (5.0)	9 (22.5)	3 (7.5)	0 (0)	0 (0)
B3: Lymphangitis (n, %)	0 (0)	0 (0)	0 (0)	0 (0)	1 (2.5)	0 (0)	0 (0)	0 (0)
B4: Systemic symptoms (n, %)	3 (7.5)	1 (2.5)	1 (2.5)	2 (5.0)	4 (10)	0 (0)	0 (0)	0 (0)
B5: Sutures removed for infection (n, %)	0 (0)	0 (0)	1 (2.5)	2 (5.0)	1 (2.5)	0 (0)	0 (0)	0 (0)
C1: Step-off border (n, %)	2 (5.0)	2 (5.0)	7 (17.5)	6 (15)	2 (5.0)	5 (12.5)	5 (12.5)	3 (7.5)
C2: Contour irregularity (n, %)	2 (5.0)	2 (5.0)	9 (22.5)	5 (12.5)	2 (5.0)	5 (12.5)	11 (27.5)	3 (7.5)
C3: Scar width >2 mm (n, %)	0 (0)	0 (0)	2 (5.0)	8 (20)	0 (0)	0 (0)	1 (2.5)	8 (20)
C4: Edge inversion (n, %)	2 (5.0)	2 (5.0)	5 (12.5)	3 (7.5)	2 (5.0)	5 (12.5)	3 (7.5)	1 (2.5)
C5: Inflammation (n, %)	0 (0)	2 (5.0)	2 (5.0)	2 (5.0)	2 (5.0)	3 (7.5)	1 (2.5)	0 (0)
C6: Overall cosmesis (n, %)	0 (0)	2 (5.0)	4 (10)	2 (5.0)	1 (2.5)	4 (10)	2 (5.0)	1 (2.5)
Hollander score (median)	6	6	6	6	6	6	6	6

The polyglactin 910 group also had a higher number of patients in comparison to the polypropylene group for parameters such as wound dehiscence (3 versus 0), wound discharge (24 versus 16), blisters (3 versus 2), stitch abscess (3 versus 1), >1 cm of cellulitis (16 versus 12), step-off border (17 versus 15), >2mm width scar (10 versus 9) and suture removal due to infection (3 versus 1). The polyglactin 910 group had a higher number of patients with systemic symptoms compared to the polypropylene group (7 versus 4). Poor overall cosmesis was observed in an equal number of patients in both suture groups i.e., 8 (20%). The median Hollander score in both suture groups was 6.An evaluation of the association of surgical outcomes i.e., wound dehiscence and surgical site infections with the suture type revealed no significant difference (Table-III). Wound dehiscence and surgical site infection were significantly impacted by female gender [WD: 1.123 (95% CI-1.003-3.149); SSI: 1.000 (95% CI-0.321-4.128)], presence of diabetes [WD: 2.000 (95% CI-1.021-4.217); SSI: 1.421 (95% CI-0.924-3.412)] and ASA Grade-III [WD: 1.241 (95% CI-1.089-3.418); SSI: 2.310 (95% CI-1.222-4.713)] (Table-IV). Patient satisfaction with wound appearance was comparable between both suture groups, with no statistically significant difference observed.

**Table-III:** Association of surgical outcomes with the suture type.

		Polyglactin 9	Polypropylene	p-value
Wound dehiscence, N (%)	Yes	2 (5.0)	0	0.452^β^
	No	38 (95.0)	40 (100)	
Surgical site infection (SSI), N (%)	Yes	2 (5.0)	0	0.459 ^β^
	No	38 (95.0)	40 (100)	
Wound healing, N (%)	Yes	38 (95.0)	40 (100)	0.521 ^β^
	No	2 (5.0)	0	

β- Chi-square/Fisher-Exact test.

**Table-IV:** Logistic regression analysis of various understudy parameters with surgical outcomes.

	Univariate					
Variable	Unadjusted odds (95% CI)	p-value	Unadjusted odds (95% CI)	p-value	Unadjusted odds (95% CI)	p-value
	Wound dehiscence		Surgical site infection (SSI)		Wound healing	
***Gender***	***Reference***		***Reference***		***Reference***	
Male						
Female	3.12 (2.127-10.962)	0.214*	2.32 (1.113-5.947)	0.120*	0.441 (0.283-7.853)	0.483
***Residence***						
***Rural***	***Reference***		***Reference***		***Reference***	
Urban	3.105 (0.185-5.957)	0.431	2.154 (0.157-9.547)	0.324	2.322 (0.192-5.401)	0.479
***Education***						
***Primary***	***Reference***		***Reference***		***Reference***	
Secondary	0.347 (0.206-6.857)	0.448	1.254 (0.957-4.598)	0.954	2.882 (0.179-8.656)	0.463
Intermediate	1.249 (0.347-5.214)	0.634	2.254 (0.115-4.741)	0.857	0.998 (0.244-3.142)	0.743
Illiterate	1.159 (0.144-3.483)	0.294	0.957 (0.641-3.542)	0.785	1.125 (0.415-2.941)	0.541
***DM***						
No	***Reference***		***Reference***		***Reference***	
Yes	0.592 (0.036-9.826)	0.224*	2.992 (1.036-4.412)	0.114*	1.689 (0.102-6.053)	0.714
***HTN***						
No	*Reference*					
Yes	1.658 (1.421-3.489)	0.198*	0.947 (1.349-3.487)	0.143*	1.242 (0.443-3.487)	0.421
***Smoking***						
No	*Reference*					
Yes	3.957 (0.254-6.318)	0.551	1.328 (0.663-4.215)	0.363	0.912 (0.112-3.127)	0.492
***ASA grade***						
I	*Reference*					
II	1.225 (0.961-4.128)	0.452	0.941 (0.229-3.428)	0.624	3.428 (0.241-5.341)	0.292
III	3.214 (1.254-4.412)	0.152*	1.253 (1.009-3.487)	0.200*	0.842 (0.123-4.334)	0.425
Age (years)	0.890 (0.729-1.085)	0.249*	0.996 (0.239-2.555)	0.202*	1.124 (0.922-1.369)	0.522
Height (cm)	0.841 (0.681-1.039)	0.108*	0.225 (0.414-4.124)	0.224*	1.189 (0.923-1.489)	0.324
Weight (kg)	1.032 (0.916-1.162)	0.606	1.624 (0.128-4.128)	0.666	0.969 (0.840-1.092)	0.731
BMI (kg/m^2^)	1.529 (0.946-2.471)	0.083	3.224 (0.441-5.413)	0.444	0.654 (0.405-1.057)	0.494
***Suture***						
Polypropylene	*Reference*					
Polyglactin 910	2.142 (0.116-4.224)	0.312	1.248 (0.314-5.129)	0.612	2.148 (0.413-6.849)	0.290
	*Multivariate*					
	*Adjusted odds (95% CI)*	*p-value*	*Adjusted odds (95% CI)*	*p-value*	*Adjusted odds (95% CI)*	*p-value*
***Gender***						
Male	*Reference*					
Female	1.123 (1.003-3.149)	0.032*	1.000 (0.321-4.128)	0.041*	1.221 (0.119-2.194)	0.412
***DM***						
No	*Reference*					
Yes	2.000 (1.021-4.217)	0.024*	1.421 (0.924-3.412)	0.039*	2.149 (0.413-5.964)	0.994
***HTN***						
No	*Reference*					
Yes	1.847 (0.419-4.743)	0.843	2.314 (0.993-6.427)	0.973	1.847 (0.843-5.449)	0.347
***ASA grade***						
I	*Reference*					
II	0.724 (0.149-5.541)	0.556	0.949 (0.124-10.259)	0.947	0.884 (0.129-7.413)	0.817
III	1.241 (1.089-3.418)	0.042*	2.310 (1.222-4.713)	0.039*	0.947 (0.222-6.423)	0.997
Age (years)	0.448 (0.119-3.419)	0.129	1.452 (0.747-5.747)	0.391	1.574 (0.999-4.781)	0.412
Height (cm)	0.192 (0.038-5.218)	0.312	2.189 (0.417-3.452)	0.841	1.174 (0.721-4.184)	0.428

## DISCUSSION

The study found that early postoperative erythema occurred more frequently with polypropylene sutures, while late complications such as wound dehiscence and discharge were more common with polyglactin 910 sutures. No statistically significant differences were found between the groups with respect to surgical site infections and cosmetic outcomes. Compared to local and international studies, our findings are in agreement with Yuenyongviwat et al.^16^ and Khan et al.^17^, with regard to similar complication rates for both suture types. In contrary to our findings, Xu et al.^18^ reported better cosmetic outcomes when absorbable sutures were used. This may have been contributed by differences in the study design, assessment of the wound, and timing of the assessment, as well as rating of the cosmetic outcomes being subjective.

The mean age in the current study was 58 years, and is lower compared to most international studies which reported a mean age of between 65 and 70 years. This may be due to the earlier onset of the disease, differences in the availability of and access to healthcare, and differences in the healthcare systems. Differences in the healthcare systems may have also contributed to higher rates of comorbidity in the current study, which included hypertension and diabetes mellitus, both of which increase the economic burden of healthcare and impact recovery from surgery.^19^ Based on the current study findings and in agreement with the reviewed literature, diabetes mellitus was found to be a significant risk factor for wound complications.

An unreported finding noted that, initially, polypropylen had a higher time-dependent difference in erythema rates, while findings showed that later, the difference was greater in polyglactin 910. This finding, which involved a reversed time-dependent difference, warrants more research in the hope of discovering more about the rates of sutures in connection to the relational time frame of the sutures to the closure and the healing of the wound. Agarwala et al.^12^ reported no dehiscence and low infection rates in total knee arthroplasty patients.

Newman et al.^20^, in their study, each reported closure of the wound by sutures resulted in the presence of superficial and deep infections. A local study, however, found closure of the wound by prolene sutures to be superior to closure of the wound by stapling, which had a greater incidence of complications.^17^ Sah et al.^21^ also found no significant disparity in orthopaedic wound complications when comparing absorbable and non-absorbable sutures.

The Hollander Wound Evaluation Scale is a validated scoring system that objectively evaluates the overall satisfaction level a patient feels toward the healing of their wound based on a score that assesses six parameters.^22^ Each of the six parameters provides a subjective measure of the healing process of the wound. In our study, the median Hollander score was the same for both suture types. However, the study of Wyles et al.^23^ was concentrated on the assessment of the perfusion of tissues, and the methods for wound closure following TKA were of secondary importance compared to the methods for scoring aesthetic outcomes of wound healing.

The assessment of the risk factors for wound dehiscence and surgical site infection was in agreement with the literature. Namba et al.^24^ showed that being a female and undergoing TKA was also a risk factor for post-operative infection, which is in agreement with the findings of our study. Several studies, including Jämsen et al.,^25^ demonstrated that being a diabetic patient is a major risk factor for a periprosthetic joint infection.

The outcomes related to wound healing that were assessed are separate and distinct. According to the CDC definition, an infection of a surgical wound is a surgical site infection (SSI). Wound dehiscence is the separation of the edges of a wound and slow or delayed healing of a wound refers to a prolonged time for a wound to heal and epithelialization of the wound without an infection or dehiscence.

### Limitations:

It includes a small sample size and short follow-up duration.

### Strengths:

It include randomized design, bilateral same-patient comparison and consistent surgical team.

## CONCLUSION

In paired (within-patient) design of bilateral total knee arthroplasty, there was no statistically significant difference between polypropylene and polyglactin 910 sutures in skin closure in postoperative wound complications, such as surgical site infection and wound dehiscence. The two suture materials proved to have equal cosmetic results. Polypropylene offers long-lasting wound care, and polyglactin 910, as an absorbent material, does not require the removal of the sutures and can be of practical use in the situations when the resources are limited. Surgical preference, patient factors, and considerations of healthcare resources should therefore inform the selection of suture material.

### Authors’ Contribution:

**MAK**: Conceptualization, study design, supervision, and final approval of the manuscript.

**NM:** Data collection, data analysis, manuscript drafting, and revision.

**MAC:** Critical review, intellectual input, and final approval of the manuscript.

**MDK:** Data interpretation, manuscript revision, and final approval of the manuscript.

All authors have approved the final version of the manuscript, and agree to be accountable for all aspects of the work.
